# Supplementary material for: The effects of BMMSC treatment on lung tissue degeneration in elderly macaques
Source: Stem Cell Res Ther. 2021 Mar 1;12:156. doi: 10.1186/s13287-021-02201-3 (PMC7923486; doi:10.1186/s13287-021-02201-3)
Supplement: Supplementary file 1 — Additional file 1. Preparation and identification of bone marrow mesenchymal stem cells of macaque [file 13287_2021_2201_MOESM1_ESM.docx]

**Preparation and identification of macaque bone marrow mesenchymal stem cells**

Two 2-3 years old macaques randomly were selected and anesthetized with an intramuscular injection of 3% pentobarbital sodium at 1 ml/kg. They were placed on the operating table in a supine position, and bone marrow aspiration was performed above and behind the anterior superior iliac spine. This was done using a 20 ml syringe containing 5 ml of heparin sodium saline (100 U / ml). Briefly, 5 ml of bone marrow were drawn. Thereafter, red blood cells were removed by the addition of 0.38% ammonium chloride to the bone marrow samples, followed by centrifugation to prepare single cells. The cells were resuspended in DMEM / F12 medium containing 20% fetal bovine serum and seeded in a 175 cm2 cell flask. The medium was changed 5 days later, and at intervals of 3 days thereafter. When the adherent cells reached became 80% confluent, they were passaged. The P4 generation of cells was used to perform morphological examination and characterization of growth patterns. Expression of surface antigens CD29, CD45, CD73, CD90, and CD184 was determined by flow cytometry. To confirm their differentiation ability in vitro, the P4 generation of cells were transferred into different special differentiation mediums to confirm their ability to differentiate into osteocytes, chondrocytes, and fat cells. The proliferation ability of cells was determined by the CCK-8 assay.

**Observation of the histological structure of lung tissues of macaque after BMMSCS infusion**

Changes in lungs were imaged with PET-CT before BMMSCS treatment and at 90 days and 180 days after treatment. Each macaque was fasted for more than 6 hours and anesthetized with 3% sodium pentobarbital at a dose of 1 ml/kg, and then blood glucose was measured. After completing the above operations, 18F-FDG was injected through the brachial vein at a dose of 3.70~4.44 MBq/kg, and the scan was performed after 60 minutes of rest. Use GE Discovery TM PET/CT Elite to scan the whole body. CT adopts conventional whole-body spiral scanning, tube voltage 120 kV, tube current 240 mA, pitch 0.561, rotation speed 0.5 s/week, layer thickness 3.75 mm, interval 3.75 mm, interval moment 512X512. After collecting image data, import the image data into the PET/CT AW4.6 post-processing workstation to calculate SUV value and Hounsfield unit.

At 180 days after BMMSCS treatment, macaque was killed by anesthesia, lung tissue was collected, observed, and photographed. The right lung was excised, fixed with 4% paraformaldehyde solution, embedded in paraffin, and sectioned. The tissue sections were used for Hematoxylin-eosin staining, we took the left peripheral lung tissues of 5 animals to make slices respectively, each slice was 0.5cm apart, and a total of 5 slices were cut for Hematoxylin-eosin staining. Then each slice randomly selected 3 fields of 400× view to observed lung tissue structure (i.e., the change of general lung anatomy, alveolar size, inflammation, alveolar septum thickness, pigmentation). Let the entire sliced tissue fill the entire field of view. Use Image-Pro Plus 6.0 software to calculate the area of each field of view (mm²) and the area of Hematoxylin-eosin staining positive area (lung parenchyma) (mm²) using the 400-fold scale as the standard. The average alveolar area (mm²) = (field of view area mm²- Area of lung parenchyma mm²)/number of alveoli. Besides, draw a cross line at the center of each field of view of each slice and Extend to the edge, then calculate the number of alveolar intervals passed by the cross line, measure the total length of the cross line (mm), and calculate the average lining interval (Mm)=total length of crosshair(mm)/number of alveolar intervals.

The tissue sections were also used for Masson's Trichrome stain. The sectioning method is the same as Hematoxylin-eosin staining. Observe under the microscope, at least two 100x fields of view are randomly selected for each slice to take pictures. When taking pictures, try to fill the entire field of view with tissue, and the background light of each photo should be as consistent as possible. Use Image-Pro Plus 6.0 software to select the measurement area by adjusting the threshold, then measure the area of blue collagen fibers. Finally, calculate the area percentage of collagen fibers.

The tissue sections were also used for Immunohistochemistry. The sectioning method is the same as Hematoxylin-eosin staining. After capturing the image, import the image to Density Quant in Quant Center. Then set the dark brown, brown, light brown, and blue nuclei of the slices to be strongly positive, moderately positive, weak positive, and negative respectively. The next step is to identify and calculate the area of strong positive, moderately positive, weak positive, and negative, and the percentage of positive. Finally, the number of positive cells in each slice and hematoxylin-eosin staining intensity are converted into numerical values for H-score scoring.
