# Supplementary material for: The effects of BMMSC treatment on lung tissue degeneration in elderly macaques
Source: Stem Cell Res Ther. 2021 Mar 1;12:156. doi: 10.1186/s13287-021-02201-3 (PMC7923486; doi:10.1186/s13287-021-02201-3)
Supplement: Supplementary file 2 — Additional file 2. Comparison of lung size between the young control group and the elderly model group [file 13287_2021_2201_MOESM2_ESM.docx]

The lung tissues from the young control group and the elderly model group were soft, butterfly-shaped, flexible, and pale red. However, the lung size of the elderly model macaques was larger than that of the young control visually.


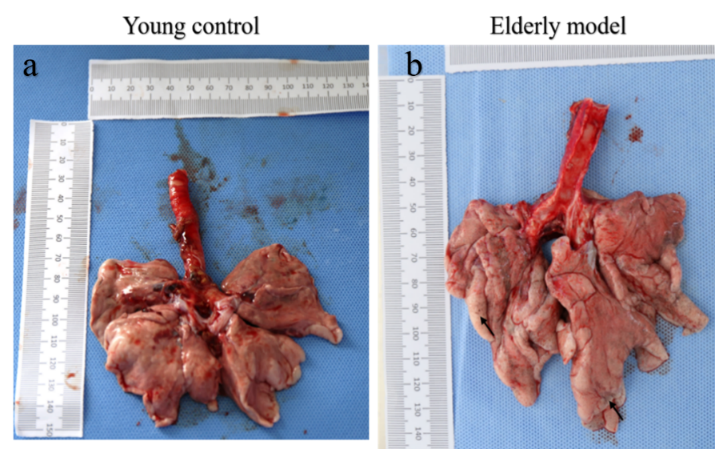


**Fig Comparison of lung size between the young control group and the elderly model group.** (a is the isolated lung of the macaques in the young control group, b is the isolated lung tissue of macaques of the elderly group)
