# Supplementary material for: The effects of BMMSC treatment on lung tissue degeneration in elderly macaques
Source: Stem Cell Res Ther. 2021 Mar 1;12:156. doi: 10.1186/s13287-021-02201-3 (PMC7923486; doi:10.1186/s13287-021-02201-3)
Supplement: Supplementary file 3 — Additional file 3. No significant changes between the treatment and the model groups [file 13287_2021_2201_MOESM3_ESM.docx]

The effects of BMMSC treatment on Lung tissue degeneration in elderly macaques

**Yu-kun YANG^▲5,1,2,3^, Ye LI^▲5,1,2,3^, Yan-ying WANG^1, 2, 3^ ,Guang-ping RUAN^1, 2, 3^ , Chuan TIAN^1, 2, 3^, Qiang WANG^1, 2, 3^, Huan-yu HE^1, 2, 3, 5^, Gao-hong ZHU^4^, Dong Fang^4^, Mao WANG^5^, Xiang-qing ZHU*^1, 2, 3^ , Xing-hua PAN*^1, 2, 3^**

**Affiliations:**

**^1^ Kunming Key Laboratory of Stem Cell and Regenerative Medicine, 920^th^ Hospital of the PLA Joint Logistics Support Force, Kunming, Yunnan Province, China, 650032**

**^2^ Stem Cells and Immune Cells Biomedical Techniques Integrated Engineering Laboratory of State and Regions, Kunming, Yunnan Province, China**

**^3^ Cell Therapy Technology Transfer Medical Key Laboratory of Yunnan Province, Kunming, Yunnan Province, China**

**^4^ Department of Nuclear Medicine, the First Affiliated Hospital of Kunming Medical University, Kunming, Yunnan Province, China**

**^5^ Kunming Medical University, Kunming, Yunnan Province, China**

**^▲^These authors contributed equally to this work**

***Correspondence to: Xiang-qing ZHU, Email: qing1021zhu@163.com, Xing-hua PAN, Email: xinghuapan@aliyun.com**

**Abstract**

Background: Age-associated lung tissue degeneration is a risk factor for lung injury and exacerbated lung disease. It is also the main risk factor for chronic lung diseases (such as COPD, idiopathic pulmonary fibrosis, cancer, among others).So, it is particularly important to find new anti-aging treatments.

Methods: We systematically screened and evaluated elderly senile multiple organ dysfunction macaque models to determine whether BMMSCs inhibited lung tissue degeneration.

Results: The average alveolar area, Mean linear intercept（MLI）and fibrosis area in the elderly macaque models were significantly larger than in young rhesus monkeys (p <0.05), while the capillary density around the alveoli was significantly low than in young macaque models (p<0.05). Intravenous infusion of BMMSCs reduced the degree of pulmonary fibrosis, increased the density of capillaries around the alveoli (p <0.05), and the number of type Ⅱ alveolar epithelium in elderly macaques (p <0.05). In addition, the infusion reduced lung tissue ROS levels, systemic and lung tissue inflammatory levels and Treg cell ratio in elderly macaque models（p<0.05）. Indirect co-cultivation revealed that BMMSCs suppressed the expression of senescence-associated genes, ROS levels, apoptosis rate of aging type Ⅱ alveolar epithelial cells (A549 cells) and enhanced their proliferation (p<0.05).

Conclusions: BMMSC treatment inhibited age-associated lung tissue degeneration.

Key words：BMMSCs, lung degeneration, type Ⅱ alveolar epithelial cells, macaque

**Introduction**

The rising aging population in China is a risk for various diseases. It is estimated that by 2050, those aged 65 years and above will represent about 20% of the Chinese population^[1]^. Age enhances structural changes of the the respiratory system, including a gradual increase in lung tissue degeneration, alveolar enlargement, alveolar wall destruction, reduced gas exchange surface area, increased airway obstruction or occlusion, decreased pulmonary vascular density, increased collagen deposition and decreased elastin, etc ^[2-4]^. Immune system disorders such as non-specific inflammation and suppressed immune responses etc ^[5]^，functional changes such as loss of elastic recoil, increased residual volume as well as gas exchange barriers^[2, 6]^ enhance lung tissue susceptibility to injury. The injuries lead to the development of chronic lung diseases such as COPD, idiopathic pulmonary fibrosis and cancer ^[7]^. Therefore, there is a need to develop options for inhibiting aging-associated lung degenerative changes.

Mesenchymal stem cells are multipotent stem cells. They are characterized by low immunogenicity, self-renewal ability, and multi-directional differentiation potential. Due to immune-associated regulation of anti-apoptosis, angiogenesis, migration and differentiation of stem cells in target organs, enhancing the growth and differentiation of local stem cells, progenitor cells and anti-scarring have been used in the treatment of various diseases^[8]^.

Studies have documented that mesenchymal stem cells can repair lung injuries and effectively treat acute and/or chronic lung diseases ^[9-11]^. However, a majority of these studies have been performed on rodents with a few of them being performed on primates. Moreover, the efficacy of mesenchymal stem cells in age-associated lung tissue degeneration has not been established.

Macaques are popular non-human primates whose biological characteristics are identical to those of humans. As model organisms, macaques are physiologically identical to humans. Their genomes have a 93% average sequence identity with humans. They are, therefore, ideal models for studies of human health and disease.

In addition, as model animals for basic and applied research in biomedicine, macaques have numerous advantages including environmental factor control and ease of scale ^[12, 13]^.

Therefore, the aim of this study was to determine the *in vivo* and *in vitro* effects of bone marrow mesenchymal stem cells (BMMSCs) on age-associated lung tissue degeneration.

**Materials and Methods**

**Animal and cell sources**

Thirty macaques aged between 2-26 years and weighing 2.2-12 kg were obtained from the Kunming Institute of Zoology of the Chinese Academy of Sciences [SCXK (Yunnan) K2017-0003]. Among them, 25 were female macaques aged between 6-26 years while 5 were male macaques aged between 2-3 years. The animals were housed in the Experimental Animal Center of the 920^th^ Hospital of the Chinese People's Liberation Army Joint Logistics Support Force, experimental animal license number: SYXK (Military) 2017-0051. Ethical approval for the use of animal models was obtained from the experimental animal council of the 920^th^ Hospital of Joint Logistics Support Force.

Bone marrow A549 cell lines were purchased form the Wuhan Sevier company, sub-cultured in a culture flask with DMEM medium supplemented with 10% fetal bovine serum. They were then incubated at 37°C in an atmosphere of 5% carbon dioxide.

**Main Reagents and antibodies**

FBS and penicillin - streptomycin solution were purchased from Servicebio; DMEM / F12 media was purchased from Hyclone; 0.25% pancreatin-0.04% EDTA was purchased from Invitrogene; 30% hydrogen peroxide solution was purchased from Solarbio; Cell senescence β-galactosidase staining kit, apoptosis detection kit, active oxygen detection kit, cell cycle and apoptosis detection kit were purchased from [Beyotime Biotechnology](https://www.baidu.com/link?url=k14pJIH-RJShldFr4KoZQFYokOaGBObCBElAnBxoC9_ZfUYX3qa87J2kdjHtcg5t&wd=&eqid=da25b16d001b46c6000000065ec54a8e); Anti-proSP-C antibody (AB3786) was purchased from Sigma; Mouse anti-human CD45 (555555), mouse anti-human CD73 (344007), Cell staining buffer, True-Nuclear Transcription Factor Buffer Set, Alexa Fluor® 647 anti-human FoxP3 (320113), FITC anti-human CD4 (317408), PE anti-human CD25 antibody (356103), PE Mouse IgG1 κ Isotype Ctrl and Alexa Fluor® 647 Mouse IgG1 κ Isotype Ctrl (400135) were purchased from Biolegend; Monkey interleukin 1β (IL-1β), monkey interleukin-17A (IL-17A) and human tumor necrosis factor alpha (TNF-α) ELISA kits were purchased from MeiMian; Fluorescent secondary antibody HRP, fluorescent secondary antibody CY3 and primers were purchased from Servicebio; GoScriptTMReverse Transcription System, and GoTaq® qPCR Master Mix were purchased from Promega Corporation; TNF alpha (7B8A11), IL-10 (20850-1-AP) and CEBPB（2B6E10）antibodies were purchased from Proteintech group; Adipogenic differentiation medium, osteogenic differentiation medium, and chondrogenic differentiation medium were purchased from Guangzhou Saiye Biological Technology Co., Ltd.; ^18^F-FDG was provided by the First Affiliated Hospital of Kunming Medical University

**Experimental protocols**

**Screening and evaluation of senile lung degeneration macaque models**

Female macaques aged between 22-26 years were used as the elderly model group, while young female macaques aged between 6-8 years old were used as the control group. A total of 5 animals were randomly distributed in each group. Five ml of peripheral blood was obtained from cynomolgus monkeys and centrifuged to obtain serum. Lung length and width were determined using a ruler. Serum samples were used to quantify TNF-α and IL-1β levels through the ELISA technique. Macaques were anesthetized with 3% pentobarbital sodium (5 ml/kg body weight) and sacrificed to obtain lung tissues. Tissue samples were divided for size, morphology and textural analysis as well as Hematoxylin-eosin staining. The Masson’s Trichrome stain was used to determine the degree of pulmonary fibrosis in the two groups. Capillary density was examined by immunohistochemistry.

**Preparation and identification of macaque bone marrow mesenchymal stem cells**

Bone marrow mesenchymal stem cells of 2-3 year old young rhesus monkeys were isolated and cultured by the adherence method. The morphology and growth characteristics of P4 BMMSCs generation cells were observed while CCK8 was used to determine their proliferative capacities. A flow cytometer was used to analyze the positive rate of their surface antigens. A special induction medium was used to induce the differentiation of P4 BMMSCs into bone, cartilage, and adipocytes. Their differentiation abilities were analyzed by staining (show in supplementary file 1).

**Cell processing and injection**

When the fusion degree of the cultured P4 BMMSCs generation was over 80%, the cells were digested and washed. They were diluted with 0.9% sterile [sodium](javascript:;) [chloride](javascript:;) [solution](javascript:;) at a concentration of 2×10 ^6^ cells/ml. After the macaques had been fixed, the BMMSCs were infused in their femoral veins at a cell dose of 1×10^7^ cells/kg per macaque, once every other day, for a total of 3 infusions. The macaques in the control and model groups were also administered with equal volumes of 0.9% sterile sodium.

**Determination of the histological structure of macaque lung tissues after BMMSCs infusion**

Lung tissue changes were determined by PET-CT before BMMSCs treatment and at 90 as well as 180 days after treatment. After 180 days of treatment, the macaques were anesthetized and sacrificed. Lung tissues were obtained and histologically analyzed (See show in supplementary file 1).

.

**Determination of the effects of BMMSCs on type Ⅱ alveolar epithelial cells**

A549 cell lines were cultured in complete mediums supplemented with 200 μmol/L, 400 μmol/L, 600 μmol/L, 800 μmol/L, 1000 μmol/L, 1200 μmol/L hydrogen peroxide. The expression levels of the P53 gene were determined by PCR while β-galactosidase staining was performed to determine the optimal hydrogen peroxide concentrations. The expression levels of *TERT, TCAB1, P53 and P21* were quantified by RT-PCR using the GoScriptTM Reverse Transcription System and GoTaq®qPCR Master Mix according to the manufacturer’s instructions. The expression of these genes reflected A549 cell senescence levels at specific hydrogen peroxide concentrations. A senescence model of type Ⅱ alveolar epithelial cell was established. Senescent cells were seeded in the lower chamber of a transwell with a pore size of 0.4 μm while an equal proportion of BMMSCs were seeded in the upper chamber. After 48 hours of co-cultivation, the expression levels of P53, P21 and TCAB1 in the A549 cells were determined by RT-PCR.

The apoptotic rate of A549 cells was determined by flow cytometry according to the Annexin V Alexa Fluor488/PI manual of 4ABIO. ROS levels and cell cycle progression were compared between the model and treatment groups using the Reactive Oxygen Species Assay Kit . Immunohistochemistry was performed to detect proSPC as markers of type Ⅱ alveolar epithelial cells. Three fields, each containing 200 cells were randomly selected after staining and used to calculate the percentage of type II alveolar epithelial cells to the total number of cells.

**Analysis of the effect of BMMSCs treatment on ROS, inflammatory factors and VEGF in elderly macaques**

Serum was isolated from the peripheral blood of macaque obtained at 0, 30, 60, and 90 days after BMMSCs treatment. Inflammatory factor (IL-1β, IL-17A, and TNF-α) levels in the peripheral blood were detected by ELISA.

After BMMSCs treatment， ROS staining was done on the left lung tissue. Sections were then subjected to the same laser intensity at equal exposure times to obtain images. To obtain the H-scores, the Densito Quant in the Quant Center was used to set dark red, brown red, light red, and blue nuclei as strong positive, moderate positive, weak positive and negative respectively. The protein levels of proinflammatory factors (IL-6, TNF-α, IL-1β) and anti-inflammatory factor (IL-10) in the lung tissues were detected by Western blot. VEGF expression levels in the lung tissues after BMMSCs treatment was determined by Western blot. ImageJ was used to analyze the gray values of all western blot images, and to compare the gray values of the internal control band to the gray values of the target protein band. The effects of BMMSCs on peripheral blood Treg cell and FOXP3 ratios in lung tissues of elderly macaques were also determined.

Lymphocytes were isolated from blood samples obtained from the animals at 0, 30, 60, and 90 days after BMMSCs treatment. Changes in Treg cell ratios in peripheral blood were detected by flow cytometry. Treg cells were labeled with FOXP3 and changes in FOXP3 content assayed by immunohistochemistry.

**Statistical analysis**

Statistical analyses were performed using the SPSS 21.0 statistical software. Data is expressed as mean ± standard deviation. Statistical differences in the means of three or more than three groups were analyzed by one-way ANOVA (One-Way ANOVA).

**Results**

**Lung tissue structures and appearance among young and elderly macaques**

Elderly macaques were found to have a dull coat that turned white, especially around the head and face. Furthermore, their skin was loose and dry while their faces appeared red (Fig. 1A).

Lung tissues of the young control group and the elderly model group were soft, butterfly-shaped, flexible, and pale red. However, lung sizes of the elderly macaque models were larger visually compared to those of the young control models (data show in supplementary files 2). The young control group exhibited clear lung structures, thin and smooth alveolar walls, no thickening of the alveolar space, no exudates, and small amounts of inflammatory cellular infiltrate around the blood vessels. In the elderly model group, the alveolar wall thickness was uniform, the alveoli were clean and without exudates, the alveolar cavity was irregularly enlarged and formed the pulmonary bullae. Furthermore, they exhibited a visible pigmentation. When compared to the young group, the elderly model group showed severe inflammation while the average area and MLI were also significantly increased in the elderly model group (Fig 1B; p <0.0001). Masson’s Trichrome stain colored the collagen fibers blue. Compared to the young control group, the collagen area in the lungs of the elderly model group was significantly increased (Fig. 1C; p<0.05). Immunohistochemical labelling of the vascular endothelial cells with CD31 revealed that the nucleus of lung tissue cells were stained blue while the surface markers of vascular endothelial cells were stained brown. In the elderly group, CD31 expression in the lungs was significantly low when compared to the young control group (Fig .1D; p<0.0001).

**Cultivation and identification of BMMSCs**

A few fusiform adherent cells were observed under an inverted phase-contrast microscope after 3-4 days of BMMSCs culturing. The cell fusion rate was 80% after 9 days of culture. BMMSCs of passage 3 to 5 exhibited a uniform morphology, were dense, spiral and isolated (Fig. 2a). To confirm the purity of the cultured cells, the P4 generation immunophenotypes of juvenile macaque BMMSCs were analyzed by flow cytometry. A panel of surface antigens was analyzed. The results showed that BMMSCs were positive for CD29, CD45, CD73, CD90 and CD184 at percentage rates of 96.35 ± 0.62, 0.16 ± 0.12, 95.22 ± 0.37, 96.25 ± 1.71, and 93.53 ± 2.76, respectively (Fig. 2b; Table 1).

Table 1: Flow cytometry analysis of surface antigens

| [Surface](javascript:;) [antigen](javascript:;) | N | Cell positive rate |
| --- | --- | --- |
| CD29 | 3 | 96.35±0.62 |
| CD45 | 3 | 00.16±0.12 |
| CD73 | 3 | 95.22±0.37 |
| CD90 | 3 | 96.25±1.71 |
| CD184 | 3 | 93.53±2.76 |

n is the number of experimental replicates

The proliferation assay showed that BMMSCs exhibited an “S” shape, were latent for the first 1-2 days, and entered a logarithmic proliferation phase in which they grew vigorously between days 3 to 7. On the 8^th^ day, they entered a plateau phase that was characterized by a reduction in proliferation (Fig. 2c). The P4 generation of young macaque BMMSCs was used to determine the *in vitro* differentiation and proliferative capacities. The duration of the differentiation experiment was 14 to 21 days. Cells were cultured in an osteogenic induction medium and allowed to aggregate, form nodules, and accumulate calcium deposits. Alizarin red stain was used to detect precipitated calcium deposits that were an indication of differentiation. Intracellular lipid droplets were stained with oil red O. Red-stained lipid droplets were found in the cells. Proteoglycans were stained with Alcian blue and appeared as smears (Fig. 2d).

**Changes in lung tissue structure after BMMSCs treatment**

Lung textures before treatment were grid-like, ground-glass opacity, honeycomb-shaped, with the peripheral, subpleural, and lower lung lobes as the main features. Emphysema was obvious. Before treatment, the average Hounsfield unit was significantly decreased when compared to the control group (p <0.01). In addition, HRCT exhibited an irregular thickening of the leaflet intervals before treatment. After treatment, the small blood vessels in the leaflet were pronounced. This was attributed to the thickening of the wall in the treatment group. PET showed that the ^18^F-FDG uptake that was quantified as the glucose uptake in the lungs decreased after treatment. At 90 and 180 days post-treatment, lung textures were clear with normal hilar. The Hounsfield unit was higher than before treatment. The average Hounsfield units were (-685 ± 12.53) and (-705 ± 18.53), respectively (Table 2; Fig. 3A).

**Table 2: PET-CT changes in elderly macaque lungs after BMMSCs treatment**

|  | n | Hounsfield Unit | SUV max |
| --- | --- | --- | --- |
| Control | 5 | -672±12.5） | 0.4±0.09 |
| [Prior](javascript:;) [treatment](javascript:;) | 5 | -853±25.3）$ | 0.7±0.06 $ |
| 90 days after treatment | 5 | -685±12.53* | 0.5±0.08 * |
| 180 days after treatment | 5 | -705±18.53* | 0.3±0.07 ** |

n is for the number of animals analyzed, $p <0.05 when compared to the control group,* p <0.05 when compared with [prior](javascript:;)

After 180 days of treatment, lung tissues appeared dark white and red without embolism. However, there were no significant changes between the treatment and the model groups (data show in supplementary file 3). Although the inflammatory score was not statistically different, the treatment group exhibited low inflammatory levels than the model group. The average alveolar area and alveolar lining interval (MLI) of the model group and treatment groups were significantly increased when compared to the control group, (p <0.05). However, the differences in the average alveolar area and alveolar lining interval between the model group and the treatment group were not significantly different (Fig.3B; p> 0.05). Masson’s Trichrome stain showed blue collagen. The collagen area of the treatment group was significantly reduced (p<0.05) when compared to the model group (Fig. 3C). To determine changes in capillary density around the alveoli after cell transplantation, immunohistochemistry was performed using CD31 as a marker of vascular endothelial cells. The nucleus were stained blue while the capillaries with CD31 surface markers were stained brown. Compared to the model group, the CD31 content around the alveoli was significantly increased in the treatment group (Fig. 3D; p<0.0001).

**Effect of BMMSCs on senile type 2 alveolar epithelial cells**

Type Ⅱ alveolar epithelium plays a significant role in lung tissue aging. Among the elderly, the quantity and quality of type Ⅱ alveolar epithelial cells are significantly reduced ^[14]^. In this study, the effect of BMMSCs on lung structure was determined using type Ⅱ alveolar epithelial cells. Hydrogen peroxide was used to establish an aging model of A549 cells. Different hydrogen peroxide concentrations were found to induce different degrees of aging in A549 cells. At 600 μmol/L hydrogen peroxide concentration, the A549 cells exhibited senescence after 6 hours of induction (data shown in supplementary file 4). This concentration was established to be the best for inducing A549 cell senescence. After the indirect co-culture of aging A549 cell model with BMMSCs for 48 hours, the lower layer of A549 cells was used to determine the effects of BMMSCs on the aging A549 cell model. The expression levels of P53 and P21 were found to be significantly decreased (p<0.001, p<0.01) in the treatment group compared to the model group. However, the expression levels of TCAB1 increased significantly (p <0.05; Fig. 4A). After indirect co-culture, the ROS level, apoptosis ratio, and A549 cell cycles were detected by flow cytometry. Compared to the model group, the ROS level and apoptosis ratio in the treatment group were found to be significantly reduced (p < 0.0001, p <0.001, respectively) (Fig. 4B, Fig. 4C). In the treatment group, proliferation was accelerated in the G2 phase (Fig. 4D; p <0.01). To verify the *in vitro* effects of BMMSCs on aging A549 cell model, proSPC was used as an *in vivo* marker for type Ⅱ alveolar epithelial cells. It was found that type Ⅱ alveolar epithelial cells were either round or oval and scattered in the alveolar wall. The number of type Ⅱ alveolar epithelial cells in the model group was significantly low when compared to the control group (p <0.001). However, in the treatment group, type Ⅱ alveolar epithelial cells were significantly elevated when compared to the model group (Fig. 4E; p<0.01).

**VEGF and TGF-β1 expression levels in lung tissues**

There were changes in the density of capillaries around the alveoli that necessitated the determination of VEGF levels in the lungs. Compared to the control group, VEGF levels in the model group were significantly low (p <0.05). After BMMSCs treatment, VEGF levels in the treatment group were significantly elevated when compared to the model group (Fig.5A; p <0.05). Since the collagen area in the lung tissue was changed after the cells had been transferred, TGF-β1 levels in the lung tissue were determined. The expression levels of TGF-β1 in the lung tissues were found to significantly increase with age. However, there were no significant changes after BMMSCs treatment（Fig. 5B）

**ROS and inflammatory factor levels after BMMSCs treatment**

Studies have revealed that the nine aging hallmarks are stem cell failure, intercellular communication changes, genomic instability and telomere wear, epigenetic changes, loss of protein homeostasis, nutrition changes, mitochondrial dysfunctions and cellular senescence ^[15]^. The main causes and impacts of these events have not been established. However, studies have documented that the causes and commonalities of these events are associated with the immune system. Inflammatory aging is characterized by elevated levels of immune cell infiltration as well as elevated levels of pro-inflammatory cytokines and chemokines in the tissue microenvironment and circulatory system ^[15]^. Under normal physiological conditions, ROS in the cells is constantly generated and eliminated. Therefore, maintaining appropriate cellular ROS levels is important for the stability of cell functions. During aging, ROS levels may also be elevated due to mitochondrial stress, damage and persistent inflammation ^[16]^. Elevated ROS levels enhance cellular damage and stimulates the immune cells to produce more pro-inflammatory factors ^[17]^. The immune regulatory and damage repair functions of mesenchymal stem cells are critical. Studies have reported that MSCs control inflammation and ROS production through paracrine and mitochondrial transfer between MSCs and aging cells ^[18, 19]^. In this study, it is shown that mesenchymal stem cells influenced lung tissue degeneration by altering inflammation and ROS levels in elderly macaques. Frozen lung tissue sections were used to detect ROS levels. An inverted fluorescent microscope showed that the nucleus of lung cells were stained blue while the cytoplasm exhibited a red fluorescence. Compared to the model group, ROS levels in the treatment group were significantly low (Fig.6A; p <0.01). To elucidate on the regulatory effects of BMMSCs on aging-associated inflammation, IL-1β, IL-17A, and TNF-α levels were detected by ELISA. Compared to the model group, IL-1β levels were found to be significantly low in blood serum (p <0.05) at 30 and 60 days after BMMSCs treatment. After 90 days of treatment, these levels had reverted to normal. Moreover, TNF-α levels were found to be significantly low after 30 days (p <0.05). However, they reverted to their normal levels after 60 days, and remained unchanged. There were no significant variations in IL-17A levels (Fig. 6B). Alterations in inflammatory factor (IL-1β, IL-6, TNF-α, and IL-10) levels in the lungs after BMMSCs treatment were determined by western blot. The levels of IL-1β, IL-6, and TNF-α in the treatment group were significantly low when compared to the model group while IL-10 levels in the model group were significantly low than in the control group (p <0.05), but were significantly elevated after BMMSCs treatment (Fig. 6C; p <0.05).

**Effect of BMMSCS treatment on immune regulatory cells**

As described above, there were changes in the expression levels of inflammatory factors in peripheral blood and lung tissues after BMMSCs treatment. Treg cells have immune-regulatory functions and play a vital role in the regulation of inflammation. Treg cell ratios in the peripheral blood were measured by flow cytometry. Compared to the normal group, it was revealed that Treg ratios in macaque peripheral blood decreased significantly at 30 days after BMMSCs treatment (p <0.01), and reached their minimum levels at 60 days after treatment (p <0.0001). Treg cell ratio changes at 60 and 90 days were not significant (Fig. 7A; p> 0.05). To determine the consistency of Treg cell changes in the periphery and lung tissues, a Treg cell surface marker (FOXP3) was used for immunohistochemical detection. FOXP3 levels of the model group lung tissue were found to be significantly elevated when compared to the control group (p < 0.01). In addition, FOXP3 levels in the treatment group were significantly low when compared to the model and control groups (p <0.0001;Fig. 7B).

**Discussion**

Age associated lung tissue structural changes are characterized by enlarged alveoli, damaged alveolar walls, decreased gas exchange surface area, increased airway obstruction or occlusion, decreased pulmonary vascular density, deepened fibrosis, and decreased elastin content ^[2]^. In this study, these phenomena were observed through pathological analysis.

Chemotaxis of mesenchymal stem cells enhances their accumulation in injured sites. These cells secrete cytokines (such as KGF, HGF) and some RNA-rich microvesicles. This paracrine effect delays cell senescence and apoptosis, lung tissue repair and inhibits fibrosis. Mitochondrial transfer and communication between MSCs and neighboring cells are involved in tissue repair ^[20-22]^.

In this study, we report on the effects of BMMSCs on lung tissue degeneration. Lung tissue changes were detected by PET-CT. There was a significant increase in x-ray transmittance (Hounsfield unit) in lung tissues after BMMSCs treatment. A significant decrease in SUV max values and fibrosis were observed. Compared to the model group, collagen deposition in the treatment group was significantly inhibited while the alveolar vascular density was significantly elevated. However, the reduction in alveolar size was not significant. These results were consistent with those reported by previous studies on mesenchymal stem cells in the treatment of COPD ^[23-25]^. Breathing is a dynamic process. Changes in the tissues around the alveoli determine the lung's inspiratory function and alveolar sizes. Due to air volume variations in the lungs during inspiration and expiration, PET-CT or Hematoxylin-eosin staining may not sufficiently evaluate whether BMMSCs can repair alveolar size deterioration during actual respiration in elderly lung tissues. Therefore, assessment of the beneficial effects of BMMSCs should be performed from multiple focal points.

SA-β-gal is a hydrolase enzyme that catalyzes the hydrolysis of β-galactosides into monosaccharides in senescent cells. P53 and its downstream genes (P21 and TCAB1) control cellular aging ^[15, 26]^.

We confirmed that BMMSCs reversed age-associated type Ⅱ alveolar epithelial cell (A549 cells) characteristics. These results were consistent with those reported in a previous study of the effects of bone marrow mesenchymal stem cells on 293T cell senescence models ^[26]^.

High inflammation and oxidative stress enhance tissue and organ degeneration during aging ^[7, 15, 27]^. In this study, we found high age-associated inflammatory and oxidative stress levels in elderly macaques. The inconsistency between the circulatory and lung tissue expression levels of various inflammatory factors differed from those previously reported in rodent lung injury models ^[28, 29]^. The fact that IL-6 was downregulated in the lung tissues after BMMSCs treatment shows that mesenchymal stem cells, such as BMMSCs, have a regenerative effect on lung tissue aging processes.

Treg cells regulate immune responses. Specifically, they play a central role in immune homeostasis and in preventing autoimmunity. They are secreted by the thymus and lymph, and transported through the [entire](javascript:;) [body](javascript:;). They inhibit the activation and proliferation of potential self-reactive T cells, thereby regulating the body's immunity and inflammation. Studies have documented that the number and functions of Treg cells change significantly in the aging body. Zhao et al reported that the ratio of Treg cells in the CD4 + cells was significantly elevated and their function was significantly inhibited in the peripheral blood of elderly mice ^[30]^. IL-10 is a multi-functional cytokine that regulates cell growth and differentiation, participates in inflammatory reactions and immune responses, and is recognized as an immunomodulatory cytokine for all innate immune cells ^[31]^. Studies have documented that the number and function of Treg cells have a regulatory effect on the IL-10 secretory roles of CD4 + and CD25+. Aging affects the capacity of CD4 (+), CD25 (+), and FOXP3FOXP3 (+) T cells to regulate IL-10 production ^[32]^. Mesenchymal stem cells are powerful immune regulatory cells that exhibit their effects on Treg cells and IL-10 production. Studies have reported that mesenchymal stem cells enhance the production of IL-10 by communicating with macrophages, B lymphocytes, and dendritic cells or by secreting PGE2, ID0, IL-6, and HO-1 ^[33]^. Treg cell levels obtained in this study were inconsistent with those reported in previous studies on the regulation of Treg cells by mesenchymal stem cells under extremely high inflammatory conditions ^[33]^. However, it was not established whether BMMSCs enhanced Treg cell tissue entry thereby inhibiting Treg ratios in the peripheral blood. FOXP3 was, therefore, used as a marker to determine Treg cell levels in lung tissues. Lung tissue findings regarding FOXP3 levels were consistent with those of the peripheral blood. However, FOXP3 levels in the lung tissues of treatment group were significantly reduced. Studies evaluating the role of MSCs in immune regulation in healthy individuals have not been documented while their effects have only been demonstrated in cell-based therapy and disease scenarios.

Interleukin 10 levels obtained in this study were consistent with those reported in a previous mesenchymal stem cell treatment model of lung injury ^[34]^. Decreased Treg cell ratios were inconsistent with the phenomenon of elevated IL-10. This implied that BMMSCs enhanced IL-10 secretion through other mechanisms other than Treg.

In summary, BMMSCs inhibit aging-associated lung degeneration. The limitations of this study were that; the impact of BMMSCs on lung functions as well as the mechanisms by which BMMSCs improved lung degeneration were not determined.

**Conclusions**

i. The elderly pulmonary degenerative macaque models revealed that the alveolar cavity was enlarged, lung structure was disordered, there was increased pigmentation, the degree of fibrosis was elevated while the capillary density was decreased.

ii. BMMSCs inhibited the degree of pulmonary fibrosis in elderly macaques, suppressed lung and peripheral blood inflammatory levels, enhanced VEGF expression in lung tissues, increased capillary density around the alveoli, and suppressed Treg cell levels in peripheral blood and lung tissues.

iii. BMMSCs suppressed the expression of type Ⅱ alveolar epithelial aging-related genes, inhibited its apoptosis, oxidative stress levels, and enhanced its proliferation. BMMSCs also elevated the number of type Ⅱ alveolar epithelium in lung tissues.

**Declarations**

- **Ethical approval and consent to participate**

Experimental protocols were approved by the Experimental Animal Ethics Committee of the 920th Hospital of the PLA Joint Logistics Support Force. The approval number: SYXK（AMY）2017-0051

- **Consent for publication**

Not applicable.

- **Availability of data and material**

All data generated or analysed during this study are included in this published article.

- **Competing interests**

The authors declare that they have no competing interests.

- **Funding**

This work was supported by grants from the Yunnan Science and Technology Plan Project Major Science and Technology Project (2018ZF007), the Yunnan Fundamental Research Projects (2017FB042）

**Authors' contributions**

YKY, YL, XQZ and XHP designed the study. YKY, XQZ, YL, QW, GPR,YYW, CT, FD, GHZ, HYH, MW carried out experiments. YKY, YL, YYW analyzed the data. YKY, YL GPR and XHP drafted and revised the paper. All authors read and approved the final manuscript for publication.

- **Acknowledgements**

We thank Freescience’s Experts for assisting with the preparation of this manuscript.

- **Abbreviations**

| **Abbreviations** | **Full name** |
| --- | --- |
| BMMSCs | Bone marrow mesenchymal stem cells |
| FOXP3 | Recombinant Forkhead Box P3 |
| IL | Interleukin |
| P53 | Tumor Protein 53 |
| P21 | Tumor Protein 21 |
| TERT | telomerase reverse transcriptase |
| MSCs | Mesenchymalstemcells |
| CCK-8 | Cellcountingkit-8 |
| ^18^F-FDG | β-2-[ 18 F]-Fluoro-2-deoxy-D-glucose |
| FBS | fetal bovine serum |
| COPD | chronic obstructive pulmonary disease |

**References**

[1] 2015—2050年中国人口老龄化趋势与老年人口预测 [J]. 中国社会医学杂志, 2018,

[2] TRAN D, RAJWANI K, BERLIN D A. Pulmonary effects of aging [J]. Current Opinion in Anesthesiology, 2017, 31(1): 1.

[3] VERBEKEN E K, CAUBERGHS M, MERTENS I, et al. The senile lung. Comparison with normal and emphysematous lungs. 1. Structural aspects [J]. Chest, 1992, 101(3): 793-9.

[4] TURNER J M, MEAD J, WOHL M E. Elasticity of human lungs in relation to age [J]. Journal of applied physiology, 1968, 25(6): 664-71.

[5] LOWERY E M, BRUBAKER A L, KUHLMANN E, et al. The aging lung [J]. Clinical interventions in aging, 2013, 8(1489-96.

[6] SKLOOT G S. The Effects of Aging on Lung Structure and Function [J]. Clinics in geriatric medicine, 2017, 33(4): 447-57.

[7] MEINERS S, EICKELBERG O, KONIGSHOFF M. Hallmarks of the ageing lung [J]. The European respiratory journal, 2015, 45(3): 807-27.

[8] XI J, YAN X, ZHOU J, et al. Mesenchymal stem cells in tissue repairing and regeneration: Progress and future [J]. Burns Trauma, 2013, 1(1): 13-20.

[9] KENNETH S, YERKOVICH S T, CHAMBERS D C. Mesenchymal stem cells and the lung [J]. Respirology, 2013, 18(3): 397-411.

[10] CUI P, XIN H, YAO Y, et al. Human amnion-derived mesenchymal stem cells alleviate lung injury induced by white smoke inhalation in rats [J]. Stem Cell Res Ther, 2018, 9(1): 101.

[11] RUBIO G A, ELLIOT S J, WIKRAMANAYAKE T C, et al. Mesenchymal stromal cells prevent bleomycin-induced lung and skin fibrosis in aged mice and restore wound healing [J]. Journal of cellular physiology, 2018, 233(8): 5503-12.

[12] SATO K, OIWA R, KUMITA W, et al. Generation of a Nonhuman Primate Model of Severe Combined Immunodeficiency Using Highly Efficient Genome Editing [J]. Cell stem cell, 2016, 19(1): 127-38.

[13] GIBBS R A, ROGERS J, KATZE M G, et al. Evolutionary and biomedical insights from the rhesus macaque genome [J]. Science (New York, NY), 2007, 316(5822): 222-34.

[14] BARNES P J. Inflammatory mechanisms in patients with chronic obstructive pulmonary disease [J]. J Allergy Clin Immunol, 2016, 138(1): 16-27.

[15] LOPEZ-OTIN C, BLASCO M A, PARTRIDGE L, et al. The hallmarks of aging [J]. Cell, 2013, 153(6): 1194-217.

[16] ROTTENBERG H, HOEK J B. The path from mitochondrial ROS to aging runs through the mitochondrial permeability transition pore [J]. Aging cell,

[17] MONICA DE LA FUENTE J M. An Update of the Oxidation-Inflammation Theory of Aging: The Involvement of the Immune System in Oxi-Inflamm-Aging [J]. Current Pharmaceutical Design, 15(26): p.3003-26.

[18] PALIWAL S, CHAUDHURI R, AGRAWAL A, et al. Regenerative abilities of mesenchymal stem cells through mitochondrial transfer [J]. Journal of Biomedical Science, 25(1): 31.

[19] HARRELL C R, SADIKOT R T, PASCUAL J, et al. Mesenchymal Stem Cell-Based Therapy of Inflammatory Lung Diseases: Current Understanding and Future Perspectives [J]. Stem Cells International, 2019, 2019(4236973-.

[20] SAVUKINAS U B, ENES S R, SJOLAND A A, et al. Concise Review: The Bystander Effect: Mesenchymal Stem Cell-Mediated Lung Repair [J]. Stem Cells, 2016, 34(6): 1437-44.

[21] LAU A N, GOODWIN M, KIM C F, et al. Stem cells and regenerative medicine in lung biology and diseases [J]. Molecular therapy : the journal of the American Society of Gene Therapy, 2012, 20(6): 1116-30.

[22] BEHNKE J, KREMER S, SHAHZAD T, et al. MSC Based Therapies-New Perspectives for the Injured Lung [J]. Journal of clinical medicine, 2020, 9(3):

[23] FIKRY E M, SAFAR M M, HASAN W A, et al. Bone Marrow and Adipose-Derived Mesenchymal Stem Cells Alleviate Methotrexate-Induced Pulmonary Fibrosis in Rat: Comparison with Dexamethasone [J]. Journal of biochemical and molecular toxicology, 2015, 29(7): 321-9.

[24] WECHT S, ROJAS M. Mesenchymal stem cells in the treatment of chronic lung disease [J]. Respirology,

[25] HUANG K, KANG X, WANG X, et al. Conversion of bone marrow mesenchymal stem cells into type II alveolar epithelial cells reduces pulmonary fibrosis by decreasing oxidative stress in rats [J]. Molecular medicine reports, 2015, 11(3): 1685-92.

[26] PAN X H, CHEN Y H, YANG Y K, et al. Relationship between senescence in macaques and bone marrow mesenchymal stem cells and the molecular mechanism [J]. Aging, 2019, 11(2): 590-614.

[27] MURRAY M A, CHOTIRMALL S H. The Impact of Immunosenescence on Pulmonary Disease [J]. Mediators Inflamm, 2015(1-10.

[28] GUPTA N, SU X, POPOV B, et al. Intrapulmonary Delivery of Bone Marrow-Derived Mesenchymal Stem Cells Improves Survival and Attenuates Endotoxin-Induced Acute Lung Injury in Mice [J]. Journal of Immunology, 179(3): 1855-63.

[29] MEI S H J, MCCARTER S D, DENG Y, et al. Prevention of LPS-Induced Acute Lung Injury in Mice by Mesenchymal Stem Cells Overexpressing Angiopoietin 1 [J]. PLoS medicine, 4(9): e269.

[30] ZHAO L, SUN L, WANG H, et al. Changes of CD4+CD25+Foxp3+ regulatory T cells in aged Balb/c mice [J]. J Leukoc Biol, 81(6): 1386-94.

[31] GABRYŠOVá L, HOWES A, SARAIVA M, et al. The Regulation of IL-10 Expression [J]. Current Topics in Microbiology & Immunology, 2014, 380(157-90.

[32] HWANG K A, KIM H-R, KANG I. Aging and human CD4+ regulatory T cells [J]. 130(8): 509-17.

[33] NAJAR M, RAICEVIC G, FAYYAD-KAZAN H, et al. Mesenchymal stromal cells and immunomodulation: A gathering of regulatory immune cells [J]. Cytotherapy, 18(2): 160-71.

[34] Activation of Human Mesenchymal Stem Cells Impacts Their Therapeutic Abilities in Lung Injury by Increasing Interleukin (IL)-10 and IL-1RN Levels [J]. Stem cells translational medicine,
