# Supplementary material for: The effects of BMMSC treatment on lung tissue degeneration in elderly macaques
Source: Stem Cell Res Ther. 2021 Mar 1;12:156. doi: 10.1186/s13287-021-02201-3 (PMC7923486; doi:10.1186/s13287-021-02201-3)
Supplement: Supplementary file 4 — Additional file 4. Hydrogen peroxide-induced senescence of A549 cells and β-galactosidase staining (100 ×) [file 13287_2021_2201_MOESM4_ESM.docx]

In this study, the effect of BMMSCS on lung structure was observed using type Ⅱ alveolar epithelial cells to explore the specific effect on lung cells. Hydrogen peroxide was used to establish an aging model of A549 cells. Different concentrations of hydrogen peroxide were found to induce different degrees of aging in A549 cells. Following SA-β-gal staining, we found that when the hydrogen peroxide concentration was 600μm/L and 800μm/L, the senescence rate of A549 cells was the highest. Besides, 1000μmol / L and 1200μmol / L were not considered as most of the cells were apoptotic and deformed.


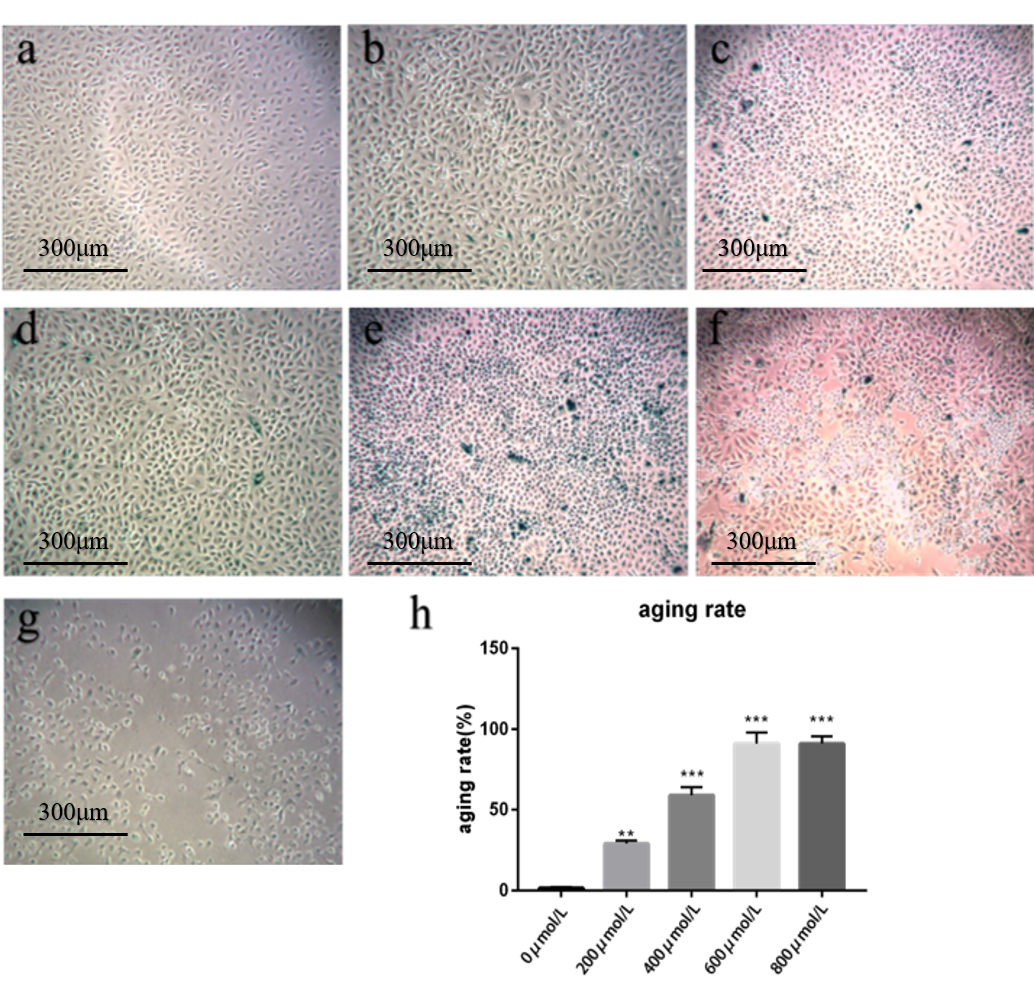


**Fig .1 Hydrogen peroxide-induced senescence of A549 cells and β-galactosidase staining (100 ×)**

a, b, c, d, e, f, g are the SA‐β‐gal staining of BMMSCS after 6 hours of induction at 0μmol/L,200 μmol/L, 400 μmol/L, 600 μmol/L, and 800 μmol/L,1000μmol/L and 1200μmol/L hydrogen peroxide concentration, respectively. h is the percentage chart of senescent cells. n=3, n is the number of repeated experiments,**P <0.01 when compared with the 0μmol/L group，***P <0.001 when compared with the 0μmol/L group)

RT-PCR was used to detect the expression of P53 gene in A549 cells after induction of aging. The increase in P53 expression was most significant (P <0.001, P <0.0001) at 600μmol / L, 800μmol / L hydrogen peroxide concentration (Fig 2A). Therefore, 600μmol/L was chosen as the optimal concentration to induce senescence of A549 cells.

RT-PCR was used to further compare the expression of P53, P21, TERT, TCAB1 before and after induction of aging using hydrogen peroxide at a concentration of 600 μmol / L. At 6h, after induction, the changes in P53 and P21 expression were significantly increased (P <0.0001, P <0.001); however, TERT and TCAB1 were significantly decreased (P <0.001, P <0.01) 6 hours after induction (Fig .2B). At 24h,48h, and 72h after changing the medium, TCAB1 did not show any significant change (P> 0.05), while P21 was significantly increased (P <0.0001). Even though the expression of P53 was significantly decreased after induction (P<0.05), it remained higher than before induction (Fig .2C).


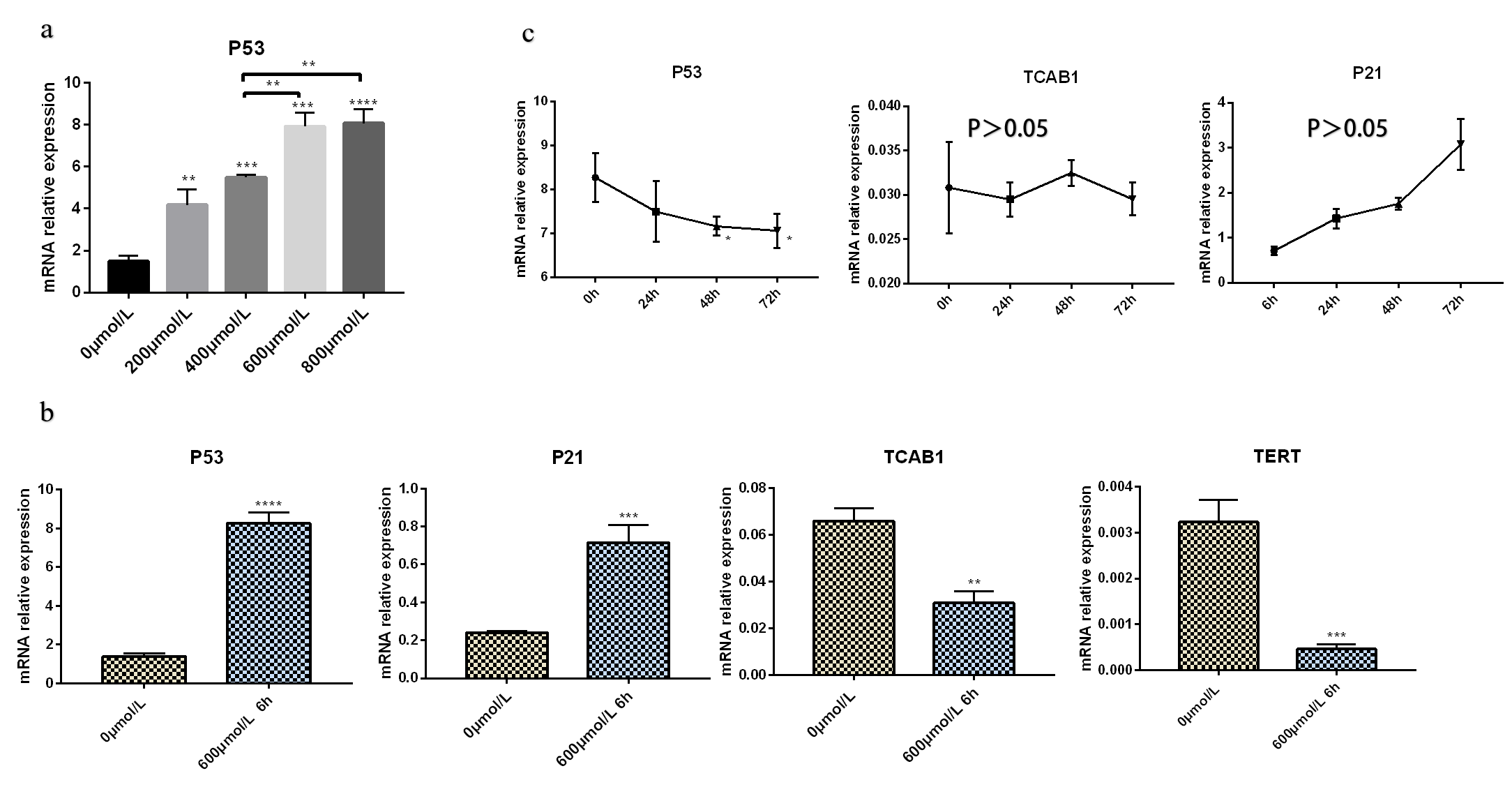


**Fig .2 Identification of A549 Aging Model a**. qPCR P53 expression After induction(n=3，n is the number of repeated experiments, &P<0.01, #P>0.05, **P<0.01 when compared with the 0μmol/L group, *** P<0.001 when compared with the 0μmol/L group, ****P<0.0001 when compared with the 0μmol/L group) **b.** qPCR validation confirming the aging characteristics of A549 cells aging model (n=3，n is the number of repeated experiments, **P<0.01 when compared with the 0μmol/L group, ***P<0.001 when compared with the 0μmol/L group, **** P<0.0001 when compared with the 0μmol/L group) **c.** qPCR validation confirming the aging stability of the A549 cells aging model (n=3，n is the number of repeated experiments, *P<0.05 when compared with the 0h group)
